# Spatiotemporal Associations between PM_2.5_ and SO_2_ as well as NO_2_ in China from 2015 to 2018

**DOI:** 10.3390/ijerph16132352

**Published:** 2019-07-03

**Authors:** Ke Li, Kaixu Bai

**Affiliations:** 1 School of Geographic Sciences, East China Normal University, Shanghai 200241, China; 2 Key Laboratory of Geographic Information Science (Ministry of Education), East China Normal University, Shanghai 200241, China

**Keywords:** PM_2.5_ pollution, SO_2_, NO_2_, spatiotemporal association, maximum covariance analysis

## Abstract

Given the critical roles of nitrates and sulfates in fine particulate matter (PM_2.5_) formation, we examined spatiotemporal associations between PM_2.5_ and sulfur dioxide (SO_2_) as well as nitrogen dioxide (NO_2_) in China by taking advantage of the in situ observations of these three pollutants measured from the China national air quality monitoring network for the period from 2015 to 2018. Maximum covariance analysis (MCA) was applied to explore their possible coupled modes in space and time. The relative contribution of SO_2_ and NO_2_ to PM_2.5_ was then quantified via a statistical modeling scheme. The linear trends derived from the stratified data show that both PM_2.5_ and SO_2_ decreased significantly in northern China in terms of large values, indicating a fast reduction of high PM_2.5_ and SO_2_ loadings therein. The statistically significant coupled MCA mode between PM_2.5_ and SO_2_ indicated a possible spatiotemporal linkage between them in northern China, especially over the Beijing–Tianjin–Hebei region. Further statistical modeling practices revealed that the observed PM_2.5_ variations in northern China could be explained largely by SO_2_ rather than NO_2_ therein, given the estimated relatively high importance of SO_2_. In general, the evidence-based results in this study indicate a strong linkage between PM_2.5_ and SO_2_ in northern China in the past few years, which may help to better investigate the mechanisms behind severe haze pollution events in northern China.

## 1. Introduction

Concentrations of fine particulate matter (denoted as PM_2.5_) in China have decreased prominently during the time period of 2013–2018 due to the implementation of the air pollution prevention and control action plan [1,2,3]. Further investigations indicate that the observed reduction of PM_2.5_ loadings in China could be largely attributed to the implementation of many effective air pollution control policies, such as fuel quality promotion and usage of cleaner energy, and especially the reduction of industrial emissions of air pollutants like sulfates and nitrogen oxides [4,5,6].

Many previous studies have evaluated the spatial and temporal variations of PM_2.5_ concentrations in China in terms of either trend estimation or spatial clustering analysis. For instance, using a global and local Moran’s I index, Ye et al. (2018) [7] examined the spatiotemporal patterns of PM_2.5_ in 338 major Chinese cities at multiple time-scales, and found that PM_2.5_ in China has evident spatial autocorrelation and clustering characteristics with “hot spots” identified in the Beijing–Tianjin–Hebei (BTH) region and southwest Xinjiang. By taking advantage of satellite-based PM_2.5_ estimations, Ma et al. (2015) [8] analyzed the spatiotemporal trends of PM_2.5_ nationwide and found that PM_2.5_ in China increased during 2004 to 2007, and has decreased since then. Similar effects were also revealed in Reference [1], but with more spatial details, as the PM_2.5_ inflection time was identified at each grid pixel. Moreover, their results indicate that performing trend analysis on stratified data (e.g., large values) is more informative and meaningful for evaluating the effectiveness of emission control actions on PM_2.5_ pollution levels [1].

Aside from trend analysis, correlations between PM_2.5_ and other air pollutants such as nitrogen dioxide (NO_2_), sulfur dioxide (SO_2_), carbon monoxide, and ozone have also been examined in many studies [4,9], aiming to explore their possible interactions in space and time [10]. Due to complex mechanisms of PM_2.5_ formation and variation, attribution of the observed PM_2.5_ variations to precursor gases via correlation analysis is by no means an easy task. Numerical simulations are always needed to achieve such a goal via sensitivity experiments, by considering various influential factors such as anthropogenic emissions and meteorological conditions [3,11,12,13,14].

Previous studies have revealed that anthropogenic emissions of SO_2_ and NO_2_ play important roles in the process of secondary fine particulate matter formation [6,15,16]. Although the newly established China national ambient air quality monitoring work has provided ample first-hand observations of six essential air pollutants on an hourly basis since 2013, few studies have attempted to examine the possible spatiotemporal associations between PM_2.5_ and SO_2_ as well as NO_2_ at the national scale using these valuable long-term in situ records.

In this study, ground measured concentrations of PM_2.5_ and SO_2_ as well as NO_2_ in China from 2015 to 2018 were used to explore possible associations between them in space and time. Differing from previous correlation analysis, maximum covariance analysis (MCA) was applied here to identify possible coupled spatiotemporal modes between PM_2.5_ and the other two pollutants. Statistical modeling practices were subsequently performed to explore the statistical relationship between them, aiming to resolve the relative contribution of SO_2_ and NO_2_ to the observed PM_2.5_ variations. Science questions to be answered by this study may include: (1) Is there any statistically significant association between PM_2.5_ and SO_2_ and/or NO_2_ in space and time across China? (2) To what extent can the observed PM_2.5_ variations be explained by SO_2_ and NO_2_? (3) Which factor is more important in modulating the observed PM_2.5_ variations?

## 2. Materials and Methods

### 2.1. Data Sources

#### 2.1.1. In Situ Air Quality Records

**Figure 1:**
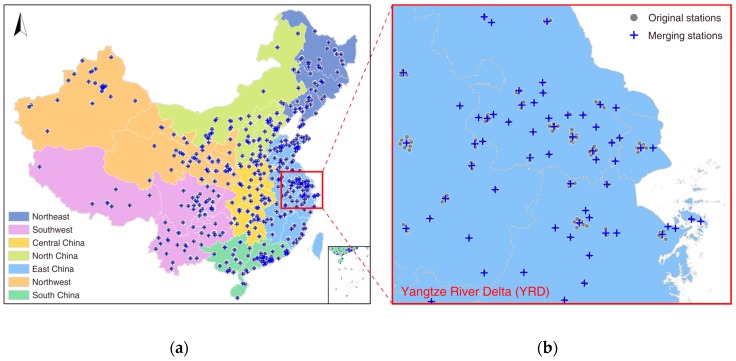
The seven major Chinese geographic divisions (**a**) and an illustrative example of spatial hierarchy clustering (**b**). Gray dots represent the original monitoring stations and blue crosses denote the aggregated stations after spatial clustering.

Hourly PM_2.5_, SO_2_, and NO_2_ concentration records during the period of 1 January 2015 to 31 December 2018, acquired from the China national environmental monitoring center, were employed. China began establishing a national ambient air quality monitoring network in 2013. To date, more than 1600 state-level monitoring stations have been deployed across 338 major Chinese cities. The spatial distribution of these stations can be found in Figure 1. Most of these stations are located in the east and south of China, where dense populations are found. Six essential air quality indicators (PM_2.5_, PM_10_, O_3_, CO, NO_2_, SO_2_) are automatically sampled at each station, primarily using a tapered element oscillating microbalance analyzer and/or beta-attenuation monitor, and are then released publicly online by the China Environment Monitoring Center on an hourly basis.

#### 2.1.2. Quality Control and Data Integration

Given observations from dense stations would not only result in significant information redundancy, but might increase the computational complexity due to significant spatial heterogeneity of air quality observations, data from 1604 stations were integrated into 530 data sets via spatial hierarchy clustering by following the process as detailed in Reference [1]. As shown in Figure 1, the clustered stations consisted of 369 merged stations and 161 independent stations.

Prior to data integration, essential quality control measures were applied to raw observations at each station to detect and remove outliers. First, a reference time series was created among stations within the same cluster using the median values of data from original stations, and a data value was referred to as an outlier once it exceeded triple (six times for SO_2_ because of large variation) the 24 h standard deviations from the reference time series. Second, gradient difference was calculated for the previously screened time series to further identify outliers. Specifically, the quadratic derivatives were calculated and data values were deemed outliers once the quadratic gradient values were not within the range of −100 to 100. Such a screening enabled identification of outliers overlooked in the previous step. Around 3.9% of PM_2.5_ observations (2.9% for SO_2_ and 5.1% for NO_2_) were identified as outliers and then removed. Quality assured hourly data values in each cluster were then averaged on a semi-monthly basis to create new time series for each air pollutant. Such a data integration scheme not only improved the data completeness in the temporal domain, but reduced the overall dimensionality of data sources. Finally, 530 clustered stations were attained, and records from only 499 stations were used for the subsequent analysis, given the presence of salient data gaps in data records from other 31 stations.

### 2.2. Methods

#### 2.2.1. MCA Method

MCA was developed to identify coupled modes between two geophysical fields by maximizing their covariance rather than correlation in the time–space domain [17,18]. Because of this unique capability, MCA has been widely applied to link two geophysical fields in time and space, especially for climate change detection and attribution, since MCA enables resolution of the coupled fields and their presentation in both spatial and temporal domains [19,20]. A detailed description of the MCA method can be found elsewhere [17].

In terms of the identified coupled modes, whether these modes are statistically meaningful is always a big concern in real practice. In this study, Monte Carlo simulations were conducted to test the statistical significance of each MCA mode by randomly permuting the time series of one field [18,21,22]. The derived MCA modes were deemed statistically significant once the actual statistics were greater than those of their random counterparts. Prior to MCA analysis, the seasonality (i.e., annual cycle) of each variable was first removed, and the goal was to avoid possible influence from local atmospheric dynamic related variations. Specifically, PM_2.5_ values at each semi-month of the year for the whole study period were averaged and then removed from the original time series. Similar procedures were also applied for SO_2_ and NO_2_ data sets. 

In principle, mapping the left and right MCA derived singular vectors geographically would reveal synergetic variations of both fields in the spatial domain. Given singular vectors always have different magnitudes, most studies have converted them to either homogeneous or heterogeneous correlation maps to better interpret the MCA results [17,23,24,25]. Homogeneous correlation maps represent the correlation values between the expansion coefficient of one field and the time series of the same field at each grid point, with another field for heterogeneous maps [26]. In this study, the spatial pattern of coupled modes were presented in the form of covariance maps, since they reveal the spatial localization of the co-varying part on each field [17,26,27].

#### 2.2.2. Quantifying the Relative Contribution of NO_2_ and SO_2_ to PM_2.5_ Variations

Although MCA enables resolution of the coupled patterns between PM_2.5_ and another two fields, it is challenging to determine which field plays a more important role in contributing to the observed PM_2.5_ variations. Statistical modeling practices were therefore performed in the present study in the form of multiple linear regression (MLR) to further evaluate the relative contribution of NO_2_ and SO_2_ to the detected regional PM_2.5_ variations. Specifically, PM_2.5_ variations and the corresponding NO_2_ and SO_2_ information were first extracted from the hot spot regions as identified in the MCA modes. Subsequently, the extracted PM_2.5_ time series were used as the dependent variable and the extracted principal components of NO_2_ and SO_2_ over the selected hot spot regions were used as explanatory variables.

The variance inflation factor (VIF) was calculated between these regressors to examine the possible collinearity problem. A threshold of 10 (an empirical value that is widely used in the literature) was also used in this study to determine the possible collinearity [28]. Finally, the LMG method [29], an advanced technique to determine the relative importance of explanatory variables in a linear model, was also applied to assess the relative contribution of NO_2_ and SO_2_ to the observed PM_2.5_ variations. A detailed description of the calculation process of the LMG measure can be found elsewhere [29].

## 3. Results and Discussion

### 3.1. Linear Trends for PM_2.5_ and SO_2_ as well as NO_2_

**Figure 2:**
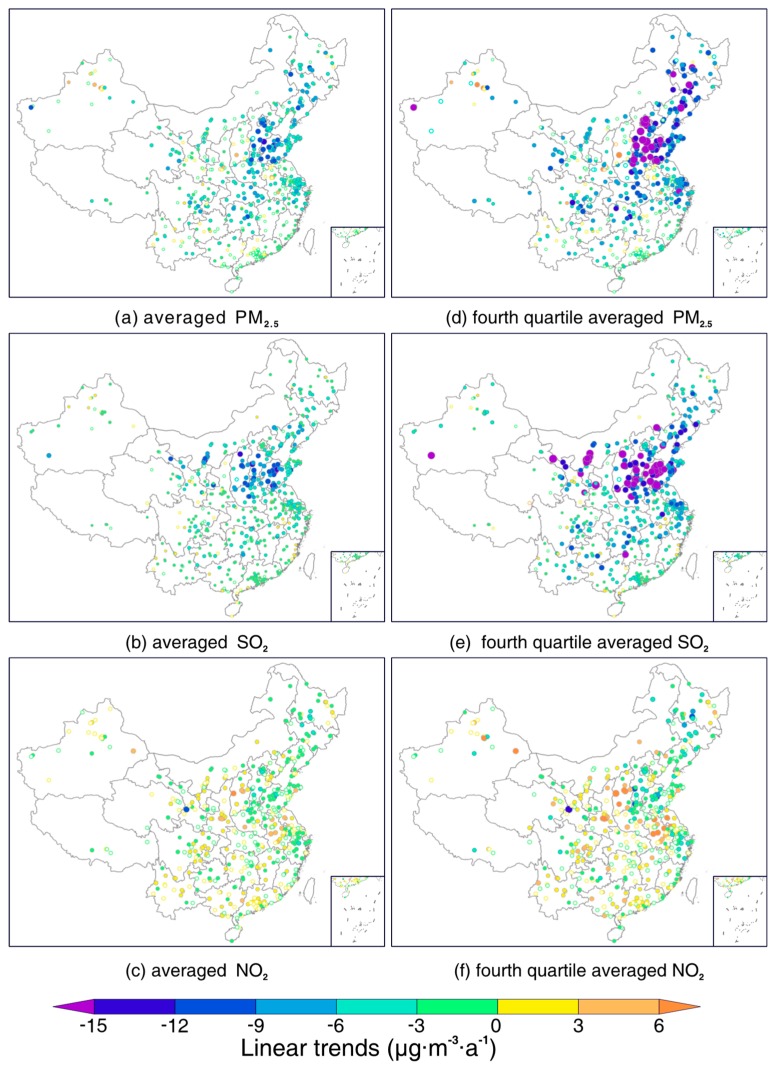
Site-specific linear trends for PM_2.5_ (fine particulate matter), SO_2_ and NO_2_ at 499 clustered stations across China from January 2015 to December 2018. (**a**–**c**) Trends derived from all data value averaged PM_2.5_, SO_2_ and NO_2_ concentrations; (**d**–**f**) trends derived from large values (averaged for values greater than the fourth quartile). Note that the radius of circles varies with the magnitudes of trend values, and the hollow circles indicate trends that failed to pass the significant test at the 95% confidence level.

To better examine possible coupled spatiotemporal variations between PM_2.5_ and SO_2_ and NO_2_, linear trends for each factor were firstly estimated using the least-square fitting method. Figure 2 shows the estimated linear trends for three factors between January 2015 and December 2018, and the left panel (Figure 2a–c) presents the trends estimated from an averaged value based on all data values, whereas the right counterparts (Figure 2d–f) were derived from their large values (i.e., averaged over values greater than the fourth quartile). It shows that PM_2.5_ and SO_2_ decreased markedly during the study time period, especially in northern China, where significant decreasing trends were observed. Moreover, in contrast to the trends estimated from all data value averages, trends estimated from large values were even more salient in northern China, indicating the effectiveness of clean air actions in reducing high loadings of PM_2.5_ and SO_2_ therein. However, no such significant declination effect was found for NO_2_ concentrations across China. In contrast, marked increasing trends (dots shaded in orange) were even observed in some regions, for example over Shanxi and Anhui provinces.

Table 1 shows the estimated linear least-square trends for these three pollutants over seven major geographic divisions (refer to Figure 1 for the geolocation of these divisions) from 2015 to 2018. It is noticeable that concentrations of PM_2.5_ and SO_2_ decreased nationwide, but had different declining rates. In regard to PM_2.5_, regions exhibiting the most significant decreasing trends included the northeast, northern China, and central China, by a rate of about −4 μg/(m^3^·a) for all value averaged concentrations and −8 μg/(m^3^·a) for large values. It shows that trends for large values almost doubled those of all value averaged concentrations, indicating the decline of PM_2.5_ loading is mainly dominated by the decreasing of severe pollution episodes. A similar effect was also observed for SO_2_, while northern China, central China, and east China were the three regions with the most significant declining trends. With respect to NO_2_, both increasing and decreasing trends were observed, whereas most trends were statistically insignificant, except for the northeast, where NO_2_ concentrations were observed with a statistically significant decreasing trend. These results collectively indicate that new actions should be implemented to control NO_2_ emissions, toward the improvement of air quality nationwide.

### 3.2. Spatiotemporal Coupled Patterns between PM_2.5_ and SO_2_ as well as NO_2_

**Figure 3:**
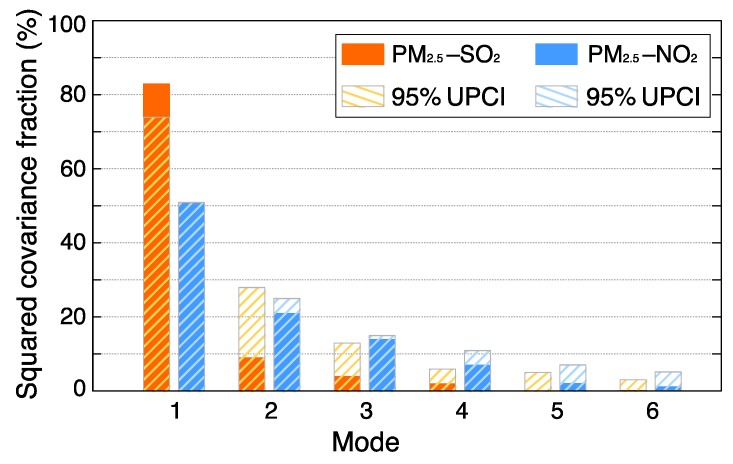
Squared covariance fraction for the first six maximum covariance analysis (MCA) modes between PM_2.5_ and SO_2_ as well as NO_2_, where 95% UPCI (upper limit of confidence interval) denotes the estimated squared covariance fraction at the 95% quantile of all values derived from the Monte Carlo simulations.

MCA was applied here to explore the spatiotemporal coupled patterns between PM_2.5_ and SO_2_ as well as NO_2_. Prior to the analysis of coupled patterns, significance of each MCA mode was firstly assessed using Monte Carlo simulations. Figure 3 shows the estimated squared covariance fraction (SCF) of each MCA mode and the estimated upper limit at a 95% confidence interval (UPCI). In principle, only MCA modes with SCFs greater than the 95% UPCI are considered to be statistically significant. Apparently, only the first MCA mode of each pair passed the significance test. In other words, statistically significant linkages could be found from the first MCA modes between PM_2.5_ and SO_2_ and/or NO_2_.

Given the significant test results, we only used the first MCA mode to explore possible coupled patterns between these three pollutants, to ensure the spatial patterns we extracted were statistically meaningful. It shows that the first MCA mode between PM_2.5_ and SO_2_ explained more than 80% of the observed covariance, indicating a tight spatiotemporal co-varying pattern between PM_2.5_ and SO_2_ across China. This effect could be also linked to the observed coincident declining trends for both pollutants. In contrast, the first MCA mode between PM_2.5_ and NO_2_ only explained about 50% of the covariance, indicating no uniform spatiotemporal variation pattern between these two factors. Given that PM_2.5_ at most stations exhibited decreasing trends across China, the small SCF values associated with coupled modes between PM_2.5_ and NO_2_ thus suggest a significant heterogeneity of NO_2_ variations nationwide. Such a deduction is also in line with the results previously shown in Figure 2, where heterogeneous NO_2_ trends were observed across China.

**Figure 4:**
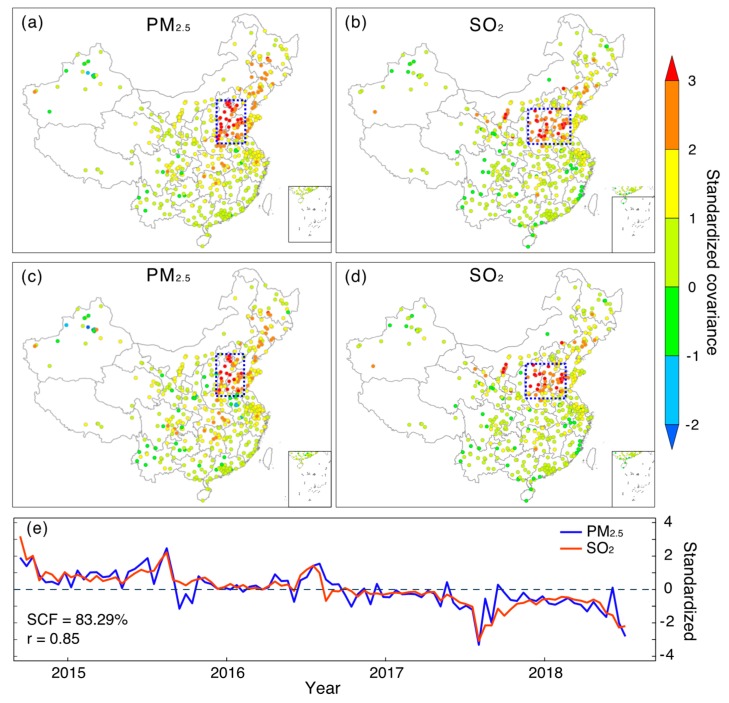
The first MCA mode between PM_2.5_ and SO_2_ coupled in the spatial and temporal domains. (**a**–**b**) Homogeneous covariance maps (covariance between expansion coefficient and time series of the same original field); (**c**–**d**) heterogeneous covariance maps (covariance between expansion coefficient and time series of another field); (**e**) standardized expansion coefficient (divided by their standard deviations) of the first MCA mode. Note that the annual cycle has been removed from each field prior to MCA analysis.

**Figure 5:**
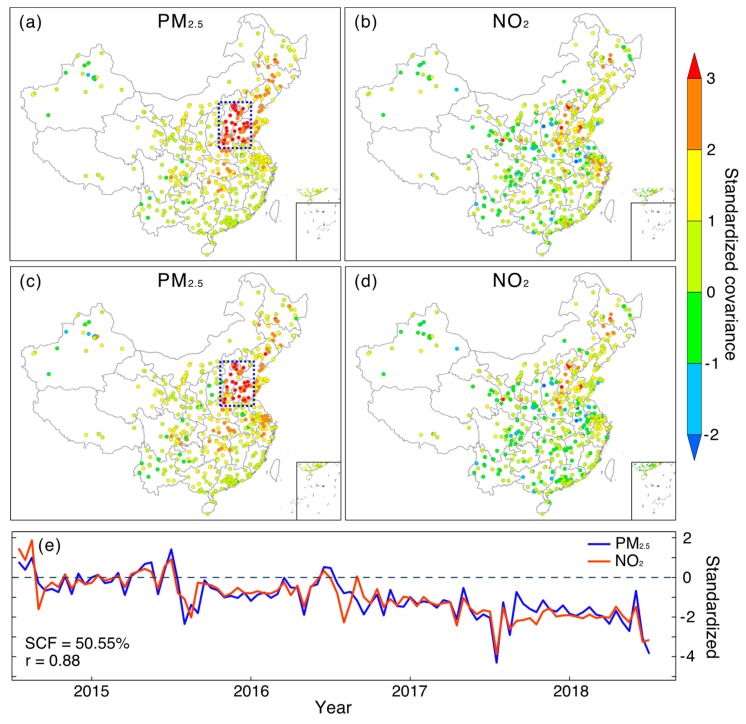
Same as Figure 4, but for coupled fields between PM_2.5_ and NO_2_. (**a**–**b**) Homogeneous covariance maps (covariance between expansion coefficient and time series of the same original field); (**c**–**d**) heterogeneous covariance maps (covariance between expansion coefficient and time series of another field); (**e**) standardized expansion coefficient (divided by their standard deviations) of the first MCA mode.

Figure 4 and Figure 5 show the first MCA modes between PM_2.5_ and SO_2_ (Figure 4) as well as NO_2_ (Figure 5), respectively. As indicated by both homogeneous and heterogeneous covariance maps (Figure 4a–d) as well as temporal variations (Figure 4e), there is an evident co-varying pattern between PM_2.5_ and SO_2_ in northern China. Specifically, the hot spot region for PM_2.5_ is mainly located over regions in BTH and the west regions of Shandong province (the outlined region in Figure 4a). With respect to SO_2_, regions exhibiting large covariance with PM_2.5_ are also observed in northern China, but cover larger areas, as Shanxi province is also included. Such a highly clustered spatial pattern in both fields indicates that PM_2.5_ and SO_2_ in these regions share a similar variation pattern in temporal domain. Moreover, it indicates a far-reaching linkage between PM_2.5_ and SO_2_ in the spatial domain, as PM_2.5_ in northern China may exhibit a similar variation pattern to that of SO_2_, even in Shanxi province. Given PM_2.5_ formations are highly associated with regional SO_2_ emissions, we may suspect that the observed PM_2.5_ variations in northern China might be modulated by not only local SO_2_ emissions, but even emissions from neighboring regions like Shanxi province. Numerical simulations could be performed in the future to corroborate this inferred linkage, more specifically, to examine whether there is an eastward transfer of SO_2_ emissions from Shanxi to northern China.

In contrast, no evident coupled field was found in the first MCA mode between PM_2.5_ and NO_2_ (Figure 5). Although a spatially localized PM_2.5_ hot spot region was identified in northern China, no such effect was found in the NO_2_ fields. Rather, stations with large covariance were observed to scatter randomly across China, indicating a lack of potent spatiotemporal coupled patterns between these two factors, regardless of the observed close correlation between their expansion coefficients (Figure 5e). In other words, the observed PM_2.5_ variation in northern China had a weak linkage with local NO_2_ emissions.

### 3.3. Relative Contributions of SO_2_ and NO_2_ to PM_2.5_ Variations in Northern China

An MLR model was established to assess the relative contribution of SO_2_ and NO_2_ to PM_2.5_ variations observed over northern China. First, PM_2.5_ anomaly time series in northern China were extracted by averaging PM_2.5_ anomaly time series at all stations located in BTH and nearby regions (i.e., hotspot regions identified from PM_2.5_ homogeneity and heterogeneity covariance maps). The first two principal components (PC1 and PC2) of SO_2_ and NO_2_ (denoted as SO_2__PC1, SO_2__PC2, NO_2__PC1 and NO_2__PC2) were then extracted over the identified SO_2_ hotspot region (outlined region in Figure 4b) and used as explanatory variables to proximate the observed PM_2.5_ variations in northern China. All regressors were standardized by subtracting their mean values and then divided by their standard deviations to make them unitless and comparable. The MLR model can be expressed as:

(1) $${\mathrm{PM}}_{2.5}\;\sim \;{\mathrm{SO}}_{2}\_\mathrm{PC}1+\;{\mathrm{SO}}_{2}\_\mathrm{PC}2+{\mathrm{NO}}_{2}\_\mathrm{PC}1+{\mathrm{NO}}_{2}\_\mathrm{PC}2$$

**Figure 6:**
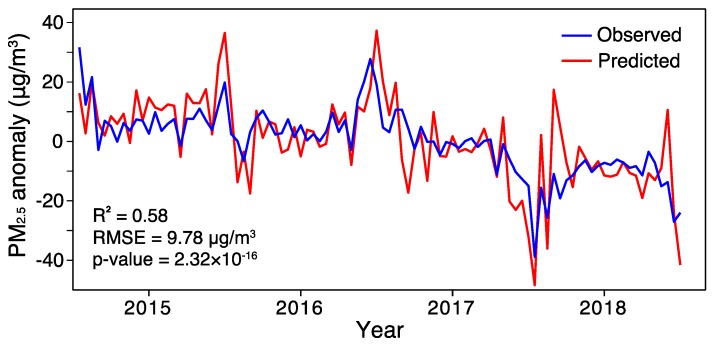
Comparison of the observed PM_2.5_ variations in northern China and the predicted one. Note that the annual cycles have been removed from each variable prior to modeling analysis.

Figure 6 simply compares the observed PM_2.5_ variations in northern China and the predicted one, using the first two principal components of SO_2_ and NO_2_. It shows that about 58% (*R*^2^ = 0.58) of the observed PM_2.5_ variances can be explained by local SO_2_ and NO_2_ variations therein. Nonetheless, the predicted time series were prone to larger oscillations than the observed one. Table 2 summarizes the regression coefficients and the estimated LMG measures for each regressor. It shows that only two regressors (SO_2__PC1 and NO_2__PC1) were statistically significant at the 95% confidence interval.

In terms of LMG measures, SO_2__PC1 had the largest LMG fraction, indicating the most significant role of SO_2_ in contributing to the observed PM_2.5_ variation. In contrast, NO_2__PC1 played a relative weak role. These effects were also evidenced by their semi-partial coefficient of determination (SPR), as the inclusion of SO_2__PC1 would result in salient improvement of R^2^ regardless of the sequence. Since the largest value of estimated VIF between these four regressors was 1.93, which is far less than the threshold of 10, we may therefore claim that the collinearity between these four regressors can be neglected. In general, the modeling results clearly revealed the relative important role of SO_2_ over NO_2_ in contributing to the observed PM_2.5_ variations in northern China from 2015 to 2018.

Nevertheless, this does not mean PM_2.5_ variations in northern China have no inherent linkage with NO_2_. Rather, there is no statistically significant linkage found between them in our cases. On the other hand, we should be aware of the fact that the temporal phase (i.e., warm versus cold or day versus night) of the relationship between PM_2.5_ and NO_2_ and SO_2_ was not accounted for in the present study, though it plays critical roles in the formation of nitrates and sulfates [30,31,32]. The effect could be explored in the future by performing MCA analysis between PM_2.5_ and day and/or night averaged NO_2_ and SO_2_ to better examine their possible linkage in space and time.

## 4. Conclusions

By taking advantage of in situ air quality records acquired from the China national air quality monitoring network, linear trends for PM_2.5_, SO_2_, and NO_2_ were evaluated for the time period of 2015 to 2018. Prior to trend analysis, data values from neighboring stations were integrated on a semi-monthly basis to improve data completeness and to reduce dimensionality. The estimated linear trends indicate salient decreasing trends for PM_2.5_ and SO_2_, especially over northern China. Moreover, trends estimated from large values almost doubled those of the all-value averaged data, indicating the need to take data magnitudes into consideration in trend analysis in order to better assess the effectiveness of clean air actions. However, no evident decreasing trend was detected for NO_2_ concentrations, even using their large data values.

The MCA results revealed an evident spatiotemporal coupled pattern between PM_2.5_ and SO_2_ in northern China from 2015 to 2018, but with different hot spot regions. Such an effect indicates a possible linkage that PM_2.5_ variations in BTH might be attributed to SO_2_ emissions, even in Shanxi province, to some degree. In contrast, no significant coupled pattern was found between PM_2.5_ and NO_2_. Further modeling practices revealed that about 58% of the observed PM_2.5_ variations in northern China could be explained by local NO_2_ and SO_2_ variations collectively. Moreover, the observed PM_2.5_ variations should be attributed largely to SO_2_ variations in northern China, given the relatively larger importance found for SO_2_ rather than NO_2_. These results collectively suggest the need to implement more stringent emission control on NO_2_ in China in the future. Given the constraint of temporal limitation of the in situ data record, a long-term study could be performed in the future by making use of satellite-derived PM_2.5_ estimations and remotely sensed NO_2_ and SO_2_ columns, measured by the Ozone Monitoring Instrument (OMI), to better explore their associations in space and time.

## Figures and Tables

**Table 1:** Region-specific linear trends (mean ± 95% confidence interval) for PM_2.5_, SO_2_, and NO_2_ from 2015 to 2018. The unit of trend is μg/(m^3^·a). Numbers in brackets after division names are the total number of clustered stations in each division.

Divisions.	Averaged PM_2.5_ (Fourth Quartile Averaged)	Averaged SO_2_ (Fourth Quartile Averaged)	Averaged NO_2_ (Fourth Quartile Averaged)
Northeast (53)	−4.55 ± 2.02 (−8.86 ± 4.85)	−3.23 ± 1.06 (−6.21 ± 1.99)	−1.19 ± 0.77 (−1.72 ± 1.35)
North China (55)	−4.15 ± 2.48 (−8.47 ± 5.14)	−4.92 ± 1.69 (−9.35 ± 3.13)	−0.13 ± 1.07 (−0.01 ± 1.72)
Central China (56)	−4.81 ± 2.24 (−7.61 ± 3.96)	−4.07 ± 0.98 (−7.74 ± 1.71)	−0.45 ± 0.91 (−0.39 ± 1.50)
East China (139)	−3.69 ± 1.75 (−6.73 ± 3.27)	−3.78 ± 0.83 (−7.31 ± 1.52)	−0.41 ± 1.00 (−0.28 ± 1.72)
South China (64)	−1.07 ± 1.32 (−2.06 ± 2.28)	−0.84 ± 0.46 (−1.87 ± 0.87)	0.26 ± 0.82 (0.53 ± 1.46)
Northwest (73)	−1.47 ± 2.28 (−3.05 ± 4.54)	−2.37 ± 1.13 (−5.45 ± 2.24)	0.33 ± 0.95 (0.75 ± 1.53)
Southwest (59)	−2.19 ± 1.33 (−3.42 ± 2.20)	−1.40 ± 0.59 (−3.10 ± 1.08)	0.24 ± 0.70 (0.48 ± 1.17)

**Table 2:** Estimated regression coefficients and LMG measures for each regressor. L-SPR (F-SPR) denotes the semi-partial coefficient of determination gains when the regressor was added as the least (first) regressor into the model. Numbers in brackets are percent variances explained by each principal component.

Regressor	Regression Coefficient	*p*-Value	LMG	L-SPR	F-SPR
SO_2__PC1 (58.26%)	8.48	1.64 $\times \;10^{-5}$	0.31	0.10	0.53
SO_2__PC2 (24.30%)	1.41	0.41	0.02	3.18 $\times \;10^{-3}$	0.03
NO_2__PC1 (30.70%)	3.33	0.04	0.21	0.02	0.42
NO_2__PC2 (24.07%)	0.20	0.92	0.03	4.72 $\times 10^{-5}$	0.03
